# 935. A Hospital Cluster of the Emerging Drug-resistant Yeast *Saprochaete clavata*

**DOI:** 10.1093/ofid/ofab466.1130

**Published:** 2021-12-04

**Authors:** Xin Hui S Chan, Cristina Cubillo-Garcia, Shanom Ali, Rebecca Gorton, Johanna Rhodes, Bennett Choy, Matthew Fisher, Kirsty Thomson, Vanya Gant, Neil Stone, Giovanni Satta

**Affiliations:** 1 University College London Hospitals NHS Foundation Trust, London, England, United Kingdom; 2 Health Services Laboratories, London, England, United Kingdom; 3 Imperial College London, London, England, United Kingdom

## Abstract

**Background:**

*Saprochaete clavata*, an ascomycetous yeast intrinsically resistant to echinocandins, is a rare yet emerging pathogen associated with invasive infections in immunosuppressed patients, particularly those with haematological malignancies. It is commonly misidentified as the closely-related *S. capitata*. Outbreaks have been associated with a high mortality rate of >50%, due in part to delayed diagnosis and resistance to commonly-used antifungals. Environmental source identification is challenging, although dishwashers and milk flasks have been implicated in previous hospital clusters.

**Methods:**

We describe a cluster of five haematology-oncology patients with disseminated *S. clavata* infections between April 2020 and April 2021 following current or recent admissions to the same ward.

**Results:**

All had prolonged (median=24 days, range=9-210) and profound immunosuppression from chemotherapy and/or stem cell transplantation for acute myeloid leukaemia (n=3) or lymphoma (n=2) at the time of culture positivity. Four were severely neutropaenic (median=0.08/mm^3^, range=0.01-0.26). Median patient age was 62 years (range=58-73). *S. clavata* was isolated from blood (n=3), urine (n=2), and liver tissue (n=1) samples. Whole genome sequencing of these isolates was performed to confirm the presence of an outbreak. All patients received empirical treatment with intravenous caspofungin before culture-guided therapy with intravenous liposomal amphotericin B +/- oral flucytosine. Two of the five patients died although both had advanced refractory malignancy. Detailed environmental sampling of fridges/freezers, drains, and vents in patient rooms and clean areas for handling or storage of food and medication failed to identify a clear point source despite isolation of multiple environmental organisms. No further cases have emerged after intensification of the cleaning regimen in these areas.

**Conclusion:**

Our experience highlights the emerging threat of drug-resistant yeasts particularly in the immunocompromised. Management of such outbreaks requires a multidisciplinary approach incorporating antifungal stewardship, infection control, and environmental microbiology, alongside close clinical liaison between haemato-oncologists and infection specialists.

**Disclosures:**

**All Authors**: No reported disclosures

